# A benchmark dataset of protein antigens for antigenicity measurement

**DOI:** 10.1038/s41597-020-0555-y

**Published:** 2020-07-06

**Authors:** Tianyi Qiu, Jingxuan Qiu, Yiyan Yang, Lu Zhang, Tiantian Mao, Xiaoyan Zhang, Jianqing Xu, Zhiwei Cao

**Affiliations:** 1 Shanghai Public Health Clinical Center, Fudan University, Shanghai, 200032 China; 2 Shanghai 10th People’s Hospital, School of Life Sciences and Technology, Tongji University, Shanghai, 200092 China; 3 School of Medical Instrument and Food Engineering, University of Shanghai for Science and Technology, Shanghai, 200093 China

**Keywords:** Bioinformatics, Pathogens

## Abstract

Antigenicity measurement plays a fundamental role in vaccine design, which requires antigen selection from a large number of mutants. To augment traditional cross-reactivity experiments, computational approaches for predicting the antigenic distance between multiple protein antigens are highly valuable. The performance of *in silico* models relies heavily on large-scale benchmark datasets, which are scattered among public databases and published articles or reports. Here, we present the first benchmark dataset of protein antigens with experimental evidence to guide *in silico* antigenicity calculations. This dataset includes (1) standard haemagglutination-inhibition (HI) tests for 3,867 influenza A/H3N2 strain pairs, (2) standard HI tests for 559 influenza virus B strain pairs, and (3) neutralization titres derived from 1,073 Dengue virus strain pairs. All of these datasets were collated and annotated with experimentally validated antigenicity relationships as well as sequence information for the corresponding protein antigens. We anticipate that this work will provide a benchmark dataset for *in silico* antigenicity prediction that could be further used to assist in epidemic surveillance and therapeutic vaccine design for viruses with variable antigenicity.

## Abstract (summary)

**Table:** 

Measurement(s)	antigenic distance • antigen
Technology Type(s)	antiserum titration value • titration
Factor Type(s)	strain

Machine-accessible metadata file describing the reported data: 10.6084/m9.figshare.12451151

## Background & Summary

Antigenicity measurements between mutated antigens are essential for the design of immunological agents for treating infectious^1^ and oncological diseases^2^. Protein antigens possessing highly similar epitopes often cross-react with the same or similar antibodies, which is commonly observed in viral pathogens such as human immunodeficiency virus (HIV)^3,4^ and seasonal influenza virus (IV)^5–8^. Additionally, the major protein antigens of viruses are continuously mutated under selective pressure. Initially, the protein antigen may maintain its antigenicity; however, the accumulation of mutations can result in antigenic escape from immune monitoring. In that case, antigenicity measurements may allow the antigenic differences among multiple protein antigens to be characterized and could further contribute to the selection or design of proper immunogens to promote a broad cross-protective immune response^9^, which is critical in the design of immunological therapeutics.

Currently, the quantification of antigenicity differences between mutated antigens relies heavily on experiments such as antibody- or antiserum-binding assays^6,10^ or the counting of amino acid mutations at essential antigenic sites. Among these experimental approaches, the HI test has traditionally been performed to determine the antigenic variations between current circulating influenza virus strains and candidate vaccines^6^. Moreover, comprehensive serological tests have been performed on both experimental animals and vaccinated or infected patients to identify the serological relationship between the subtypes of Dengue virus (DENV)^11^. Typically, immunological experiments require extensive antibody or antiserum preparation, dilution, and standardization. Thus, computational algorithms for estimating the antigenic distance between multiple protein antigens in a high-throughput manner are highly desired. Regarding *in silico* approaches, there have been multiple efforts aimed at antigenic distance prediction between influenza vaccines and circulating strains by generating theoretical models based on the sequence or the structure of antigen proteins. For instance, the mutations between two antigen proteins were counted at antigenic sites^12,13^, and the numbers of mutations were correlated with the experimental distance^14,15^. Additionally, structural features could be derived from antigen proteins to establish an antigenicity prediction model based on the spatial context of the antigenic sites^16^. The collection of sequences and the experimental dataset could be important for the detection of mutations and the design of sequence-based and structure-based antigenicity prediction models. However, the construction of *in silico* methods is still a great challenge due to the lack of standard benchmark datasets.

To construct an *in silico* model, a benchmark dataset should include two major components for antigenicity measurement: *(i)* sequence or structure information for protein antigens and *(ii)* the experimentally validated quantitative or qualitative antigenic relationship between the two protein antigens being compared. Then, statistical models, machine learning models, or deep learning models can be used to establish rapid computational tools for quick and accurate antigenicity prediction. In this paper, we present collated and annotated benchmark datasets for (1) haemagglutinin (HA) sequences of influenza A virus (IAV) A/H3N2 and influenza B virus (IBV) with standard HI-test results and (2) envelope protein sequences of DENV with antiserum neutralization experiments. All antigen pairs collated in this benchmark dataset were annotated with quantitative or qualitative antigenicity relationships based on HI-test experiments or titration data from antiserum experiments. A portion of the data from the benchmark datasets was previously used to establish antigenicity measurement models for emerging pathogens such as influenza viruses^16^ and Dengue viruses^9,17^. Given the extensive scope of antigenic clustering^9^, vaccine failure detection^16^ and broad-spectrum vaccine design^9^, the benchmark datasets presented here could guide the development of *in silico* approaches for antigenicity monitoring and the selection of potential broad-spectrum vaccines.

## Methods

### Structure of the benchmark data for antigenicity measurement

**Fig. 1:**
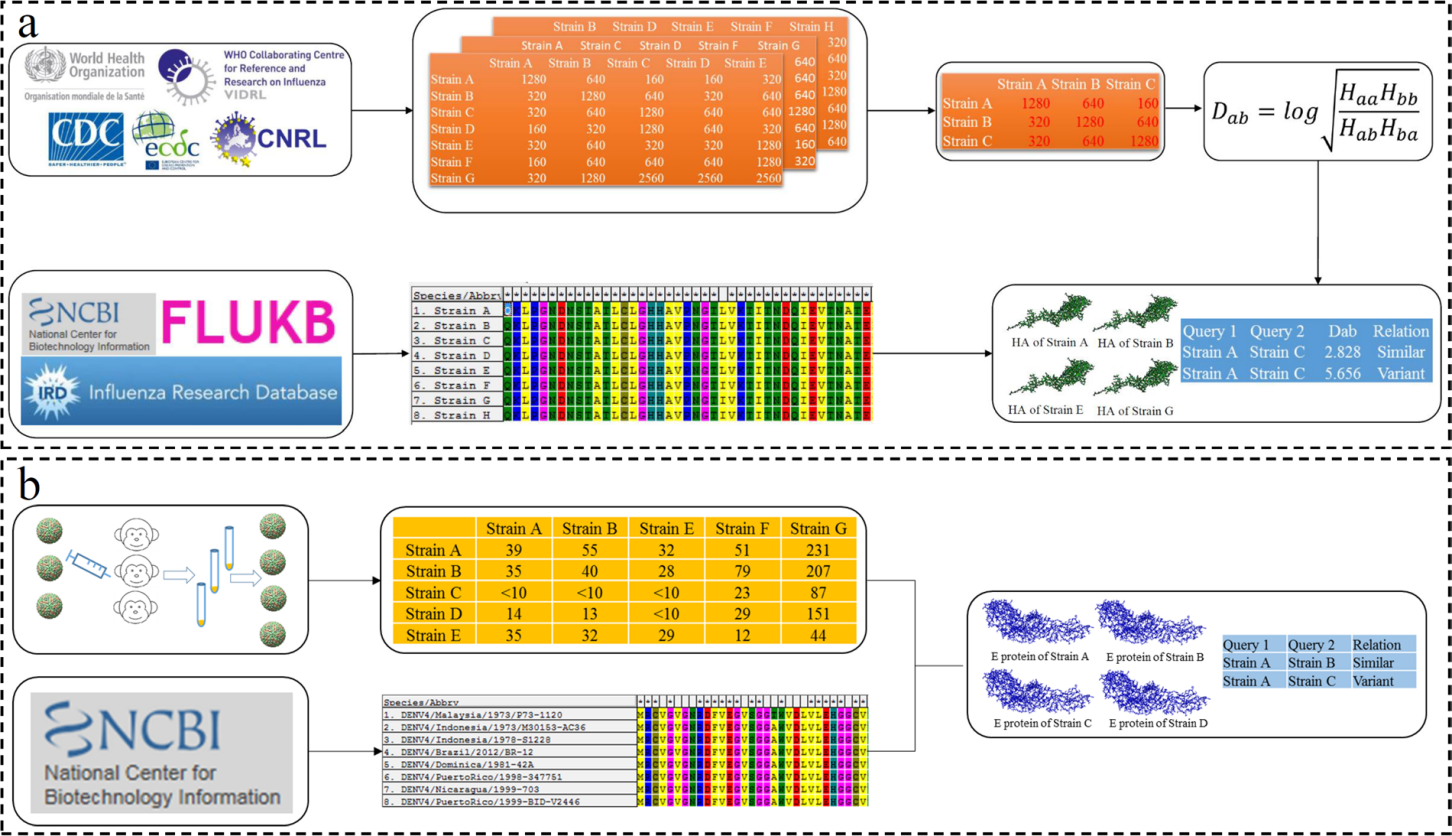
Illustration of benchmark data collection. (**a**) Benchmark data for influenza virus. The HI-test data for both IAV A/H3N2 and IBV were collected from reports of international organizations and published articles with pre-processed antigenic distances. The sequence data of HA proteins were collected from multiple virus databases. (**b**) Benchmark data of DENV. Antisera data were collected from African green monkeys, and envelope protein sequences were collected from NCBI virus databases.

The benchmark dataset for antigenicity measurements required two components: (*i)* antigen proteins with sequence information and *(ii)* the experimentally verified antigenic distance between the two compared antigen proteins. The antigenic distance determined in experiments such as the HI-test or calculated from antiserum data is preferable for benchmark data. For instance, multiple international organizations provide weekly or annual reports on influenza epidemic surveillance based on evaluating the antigenicity variations of circulating strains through the HI test. The HA sequences of the corresponding strains involved in the HI test were collected from virus databases including the National Centre for Biotechnology Information (NCBI) database^18^, FluKB^19^, and IRD^20^. Furthermore, the antigenic relationship between the two compared antigens can be defined by dilution values in the HI test **(**Fig. 1a). Similarly, samples were collected from African green monkeys for experimental titration for DENV antigenicity evaluation^11^. Envelop protein sequences from the corresponding strains were derived from virus variation resources of the NCBI^21^ (Fig. 1b).

### Benchmark dataset of influenza virus haemagglutinin

The HI assay values for influenza viruses, including IAV A/H3N2 and IBV, were obtained from reports of international organizations and published articles^22–33^, which were the gold standard for antigenic measurement between influenza viruses. The antigenic distance (*D*_*ab*_) between strains *a* and *b* was analysed by introducing all four individual haemagglutination-inhibition titres (*H*_*aa*_, *H*_*ab*_, *H*_*bb*_, *H*_*ba*_) and was defined as follows^34^:

1 $${D}_{ab}=log\sqrt{\frac{{H}_{aa}{H}_{bb}}{{H}_{ab}{H}_{ba}}}$$

where the *H*_*ab*_ HI titre represents the maximum dilution of serum raised against strain *a* that is necessary to inhibit cell agglutination caused by strain *b*. Two viruses were defined as antigenic variants when the *log*^−1^*D*_*ab*_ value was above 4; otherwise, they were considered antigenically similar^14^. For a given strain pair, the HI test may produce different results due to the different experimental conditions of each study. To the experimental differences, for the HI values of a given strain pair derived from different resources, the $\left|{D}_{ab}-\bar{{D}_{ab}}\right|$ values within the top 10% were removed in descending order ($\bar{{D}_{ab}}$represents the average value of *D*_*ab*_). Next, the average value of the remaining *D*_*ab*_ values was calculated as the antigenic distance between strain *a* and strain *b*.

The haemagglutinin sequences of IAV A/H3N2 and IBV were collected from international databases, including the influenza virus resource of the NCBI^18^, FluKB^19^, and IRD^20^. For quality control, HA sequences with alignment lengths longer than 327 amino acids for IAV A/H3N2 and IBV were retained. Finally, the results of HI assays and the corresponding HA sequences of IAV A/H3N2 and IBV were generated as benchmark datasets. For IAV A/H3N2, 3,867 strain pairs with 2,286 antigenic variant pairs and 1,581 antigenically similar pairs were included. For IBV, 559 strain pairs with 274 antigenic variants and 285 antigenic similarities remained. These methods are expanded versions of those used in our previous work^9,16^.

### Modelling the antigenic variance for IBV

To construct the antigenic measurement model for IBV, the dominant antigenicity-related positions were first identified. Based on multiple sequence alignment, all haemagglutinin sequences of IBV were mapped to the full alignment length of 327 amino acids. For any two compared IBV strains, if an aligned position contained the same amino acids, it was marked as 0; otherwise, it was marked as 1. Thereafter, a 327-bit binary descriptor could be generated, and the antigenic distance between two compared strains was used as a classification indicator.

Furthermore, positions that were closely related to the antigenic variants were derived through a linear regression model. Through 10-fold cross-validation, positions with a weight |ω| > 0 were selected as antigenicity-dominant positions. After identifying the antigenic-dominant positions, machine-learning approaches including the naive Bayes, logistic regression, simple logistic, and random forest methods were introduced to generate *in silico* models for IBV.

### Calculating the antigenicity coverage of the vaccine strain

To calculate the antigenicity coverage of WHO-recommended vaccine strains, 11,419 HA1 sequences of IBV with an aligned length of 327 amino acids recorded from 1959 to 2016 were derived from the influenza virus resource of the NCBI. Based on a sequence similarity of 99.3% identity, 389 non-redundant HA sequences were retained as representative proteins for further analysis. Then, the antigenicity coverage of each WHO-recommended vaccine strain in each year was defined according to Eq. 2:

2 $${C}_{{i}_{Y}}=\frac{{S}_{Y}}{{N}_{Y}}$$

where ${C}_{{i}_{Y}}$ represents the coverage of vaccine *i* in year *Y*, *N*_*Y*_ represents the total number of emerging strains collected in year *Y*, and *S*_*Y*_ represents the number of antigenically similar strains for vaccine *i* in year *Y*.

### Benchmark dataset of the Dengue virus envelope protein

Envelope (E) protein sequences of Dengue viruses (DENV) were collected from the NCBI virus variation database^35^. For quality control, the E protein sequences of DENV serotypes 1 to 4 with aligned lengths over 495 amino acids were retained.

Antiserum titrations of DENV were obtained from neutralization assays conducted in experiments on the African green monkey by Katzelnick *et al*.^11^. After removing strain pairs without labels (empty value) and setting all values “<10” as 5 to simplify the calculation, a total of 1,072 strain pairs with antisera values were retained. All titre values were normalized to 0–1 by setting the highest normalized value in each row as 1. Normalized titre values could be obtained with the following equation:

3 $${V}_{n}=\frac{V}{{V}_{max}}$$

where V_n_ represents the normalized titre value, V represents the original value in the titre table, and V_max_ represents the maximum value of the original titre value in each row. These methods are the expanded version from our previous work^9^.

## Data Records

**Fig. 2:**
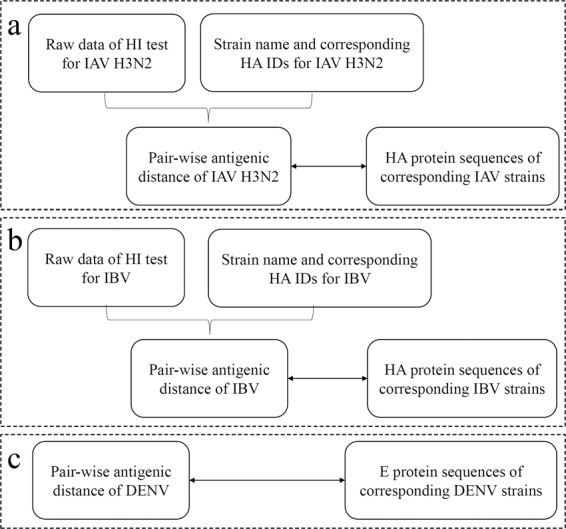
Data records of the benchmark dataset. (**a**) Data records of the HI values and haemagglutinin sequences of IAV A/H3N2. (**b**) Data records of the HI values and haemagglutinin sequences of IBV. (**c**) Data records of the neutralization titre values and E protein sequences of DENV.

The benchmark dataset for antigenicity measurement contained three major components: (1) HA protein sequences of IAV A/H3N2 with antigenic distances determined via the HI-test for corresponding strain pairs, (2) HA protein sequences of IBV with the HI-test results for corresponding strain pairs; and (3) envelop protein sequences of DENV with antigenic distances determined from the antisera titration tests in a previous study^11^. Detailed information on the data structures is illustrated in Fig. 2. All data are available at figshare (10.6084/m9.figshare.c.4961501)^36^.

### Data records for IAV A/H3N2

As illustrated in Fig. 2a, the raw data from the HI tests of IAV A/H3N2 isolates obtained from multiple reports are listed in a “Summarized HI tables.csv” file of all raw data obtained from different resources and a file named “Summary-table.xlsx” with detailed information, which includes the strain name, accession or citation information, and data record. Additionally, strain names and corresponding HA IDs are provided in “Strain_HA for IAV.txt”. Furthermore, the ratios of the pairwise antigenic distance and the *D*_*ab*_ values of two compared strains derived from historical experiments are provided in the “HI-test value for strain pairs.csv” file, and the corresponding sequences of haemagglutinin proteins are recorded in the “Sequence data for influenza A H3N2.fasta” file.

### Data records for IBV

Similarly, the summary of the HI tables of IBV is provided in “HI_total for IBV.csv”, and the strain names and the corresponding HA IDs for IBV are provided in “Strain_HA for IBV.txt”. The pairwise antigenic distances of the *D*_*ab*_ values between two compared strains for IBV (pairwise antigenic distance for IVB) and the corresponding sequences of the haemagglutinin proteins are listed in the “HA sequence data for IBV.fasta” file (Fig. 2b).

### Data records for DENV

As illustrated in Fig. 2c, the raw data on antiserum titres were derived from Table S3 of Katzelnick *et al*.’s work^11^, and the normalized values are recorded in “Normalized titer for DENV.xlsx”. The corresponding envelope protein sequences of Dengue virus serotypes 1–4 are listed in the “Sequence data of DENV.fasta” file.

## Technical Validation

### Detecting antigenic drift in emerging pathogens

To explore the potential utility of the benchmark dataset for the monitoring of new antigenic clusters, we evaluated the antigenic clustering and antigenic drift events of IAV A/H3N2 over the past four decades based on our benchmark dataset. The antigenic distance between all prevalent strains from each year was calculated with CE-BLAST^9^^,^ and the dominant strain for each cluster was selected according to chronological order. Initially, the dominant strain of the year 1968 was set as A/Hong Kong/1/1968, and the dominant strain of the following year was identified as the variant with the highest antigenic coverage in the circulating year. A new antigenic cluster arises only when the antigenicity coverage of an antigenic variant strain in the circulating year is substantially greater than the coverage of the dominant strain from the previous year. If an antigenic variant strain becomes the dominant strain in the circulating year, a new antigenic cluster is generated; otherwise, the current antigenic cluster remains.

For year *Y* with *N* strains, the antigenic distance was calculated between all strain pairs in our dataset. The antigenic coverage of strain X was defined as ${C}_{X}=\frac{{N}_{X}}{N}$, where *N*_*x*_ represents the number of antigenically similar strains (antigenic distance < 4) of strain *X* in the circulating year. The initial year (*Y*_0_) was set as 1968, and the dominant strain (*X*_0_) was set as A/Hong Kong/1/1968. For the next year, *Y*_1_, if strain *X*_1_ has antigenically drifted from strain *X*_0_ (antigenic distance > 4) and exhibit a sufficiently high antigenicity coverage (*C*_*X*_ > 30%) in year *Y*_1_, it will become the dominant strain in year *Y*_1_ and will be defined as the representative strain of a new cluster. Otherwise, the dominant strain of year *Y*_1_ is defined as strain *X* with the highest antigenic coverage in *Y*_1_ and remains in the same cluster as in the previous year.

**Fig. 3:**
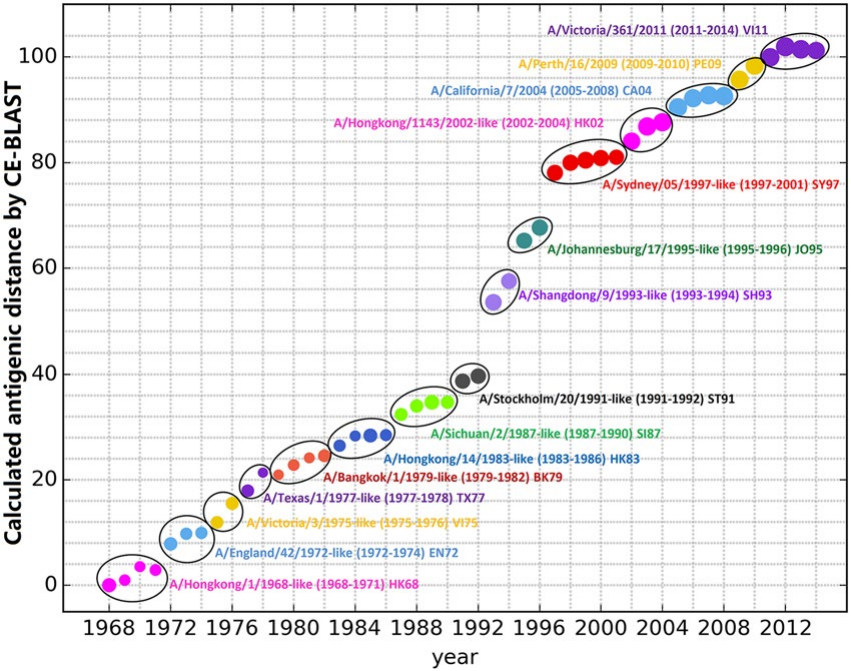
Antigenic clustering over the past four decades (1968–2014). The X-axis illustrates different years, while the Y-axis illustrates the predicted antigenic distance. Each spot represents the dominant strain of the circulating year, whose size is proportional to the logarithm of the strain numbers in that year. Strains with similar antigenicity are grouped into one antigenic cluster and named according to the first dominant strain in the first year of the cluster. Within each cluster, the antigenic distance was calculated between the dominant strain of each year and the representative strain of the cluster, whereas the antigenic distance between the two neighbouring clusters was calculated based on the representative strain.

Next, the antigenic mapping of 16,672 historical strains was performed based on the antigenic distance calculated with CE-BLAST^9^. In Fig. 3, different antigenic clusters are indicated in different colours. During the 47 years from 1968 to 2014, 14 antigenic drift events were identified. These results agree well with the experimental study of Smith *et al*.^6^. and the *in silico* prediction study of Du *et al*.^37^. In Smith’s work^6^, 11 antigenic clusters were experimentally determined based on 273 viral isolates and were named after the first vaccine strain of that period. As shown in Fig. 3, all 11 experimentally identified representative vaccine strains were placed in distinct clusters. Moreover, an additional antigenic cluster represented by the A/Hong Kong/14/1983 strain from 1983 to 1986 was also detected. Although the experiments failed to detect this cluster, the large-scale antigenicity mapping performed in Du’s work indicated its existence^37^. In this study, 15 antigenic clusters were determined from 1968 to 2010 based on the antigenic predictions for 1,071 HA sequences. Among these clusters, 13 were consistent with our discoveries according to each period, whereas two clusters, represented by CA04 and JX06, were grouped into one cluster in our results. The high concordance between the two experimental antigenicity mapping and large-scale *in silico* prediction analyses illustrates the usefulness of our benchmark dataset and expands the utility for antigenic monitoring in our related works^9,16^.

### Monitoring antigenic coverage for vaccine strains

For emerging pathogens such as IBV, the WHO proposes vaccine strains for the coming season that are predicted to provide wide protection against the majority of the circulating strains during the valid time period^38^. Frequent mutations in the main proteins of emerging pathogens may lead to antigenic drift and cause vaccine failure. Vaccine strains that fail to cover the majority of circulating strains should be replaced by another strain with higher antigenic coverage. Thus, one of the key issues in vaccine selection relies on the evaluation of antigenic distance, which was obtained via HI assays between proposed vaccine strains and selected circulating strains in the present study. Here, we are trying to quantitatively estimate the antigenic coverage of WHO-recommended vaccine strains and evaluate the potential utility for monitoring the efficiency of the vaccine strains.

**Fig. 4:**
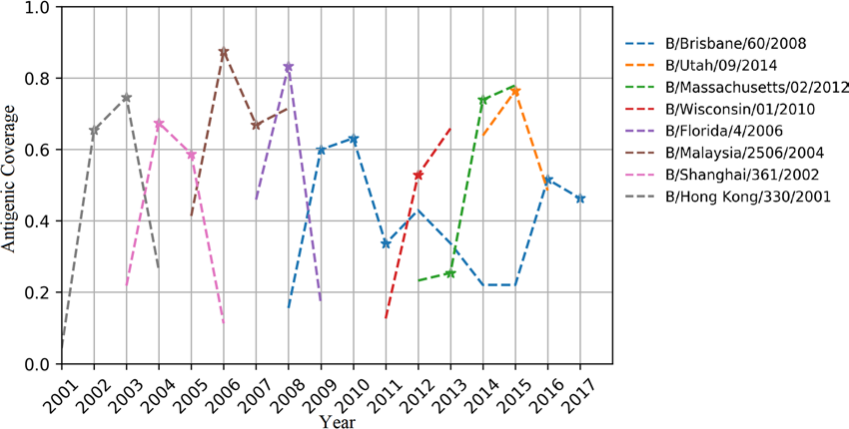
Vaccine coverage in the Northern Hemisphere from 2001 to 2017. The X-axis represents years from 2001 to 2017, and the Y-axis represents the antigenic coverage of vaccine strains in each year. Each line refers to a vaccine strain from the year before it was proposed as the vaccine strain to the year after it was replaced by updated vaccine strain. Stars indicate the years in which each vaccine strain was recommended.

To calculate the theoretical antigenic distance between multiple strains, the *in silico* model for IBV was constructed based on our benchmark dataset (*see Methods*). Here, the best prediction model constructed based on the random forest classifier was used for further analysis. With this model, the theoretical antigenic distance could be dynamically calculated between each WHO-recommended vaccine strain against all available strains circulating in the northern hemisphere from 2001 to 2017. Typically, the antigenic coverage of a newly emerging strain will be low when it is not the dominant circulating strain. Then, strains with increasing antigenic coverage will be selected as vaccine strains for several years and will later be replaced when new dominant strains arise. The antigenic coverage of WHO-recommended vaccine strains for IBV is illustrated in Fig. 4; most of the vaccine strains could successfully cover the antigenicity of over 50% of the circulating strains, and the curve of antigenic coverage displayed an inverted-V distribution, with an ascending-maintaining-descending shape. More interestingly, strain B/Brisbane/60/2008 was recommended as the vaccine strain in 2009–2011 before it was replaced by other strains. However, it was recommended again as the vaccine strain for 2016 to 2017. These results agree well with our monitoring results, according to which strain B/Brisbane/60/2008 presented an “M”-shaped curve with two peaks, in 2009–2010 and 2016–2017. Thus, based on the benchmark dataset, it is possible to monitor the antigenic coverage of each circulating strain, and this approach provides the potential to propose effective vaccine strains for the coming season.

## Usage notes

Understanding the antigenicity differences between protein antigens is essential for the development of immunological therapeutics. Thus far, the accumulation of protein sequences, the spatial structures of antigens, and the obtained experimental results have largely facilitated the identification of antigenic determinations. Currently, the estimation of antigenic variations based on *in silico* models remains a great challenge because of the lack of a large-scale benchmark dataset. In this study, we systemically collated three essential benchmark datasets for antigenicity measurement, including (1) HA sequences of IAV A/H3N2 with antigenicity relationships derived from historical HI-test values, (2) HA sequences of IBV with antigenicity relationships derived from historical HI-test values, and (3) animal titre values of DENV serotypes 1–4 with antigenicity relationships derived from previous studies^11^. All benchmark datasets were collated and normalized according to the procedures described above to ensure the quality of the antigenicity measurements. For approaches that require protein structure information for antigenicity prediction, the sequence data could be modelled through the available homology modelling approach^39^ before model construction (Supplementary Note). The feasibility of using homology-modelled structures for antigenicity calculation is evaluated in the Supplementary Note. We expect that the benchmark datasets presented here will be useful for (1) constructing a computational model for high-throughput antigenicity measurement^9,37^, (2) epidemic surveillance of infectious diseases^9^, (3) effectiveness monitoring of vaccine strains^16^, (4) antigenicity clustering analysis of emerging pathogens^9,17^ and (5) broad-spectrum vaccine design^9^. All the above benchmark datasets have been deposited in Figshare^36^.

## Supplementary information

Supplementary Note

## Data Availability

Data pre-processing tools for (1) pre-determining epitope and paratope residues, (2) re-numbering antibody residues with numeric identifiers, and (3) re-labelling multiple chains have been uploaded to GitHub at https://github.com/baddtongji/CE_BLAST. The methods involved in the ***technical validation*** are integrated into the CE-BLAST web server and can be accessed at http://bidd2.nus.edu.sg/czw/ce_blast/.
