# Supplementary material for: A benchmark dataset of protein antigens for antigenicity measurement
Source: Sci Data. 2020 Jul 6;7:212. doi: 10.1038/s41597-020-0555-y (PMC7338539; doi:10.1038/s41597-020-0555-y)
Supplement: Supplementary file 1 — Supplementary Note [file 41597_2020_555_MOESM1_ESM.docx]

**Comparison of crystal structures with homology modelling structures**

Since B-cell epitopes are mostly conformational epitopes with spatial structures, *in-silico* models which could incorporate the structure information of protein antigens could better characterize the antigenicity variation. Among the massive data of protein sequences, only a small part of antigens contains resolved crystal structures and the rest proteins require homology modelling to construct the conformational structures. To test the capability of modelling structures for antigenicity measurement, the hemagglutinin protein of IAV was chosen as an example. A total of 21 HA crystal structures derived from PDB^1^ were labelled as group C. Homology modelling structures of corresponding HA proteins were constructed through Modeller 9.11^2^ and labelled as group M. Furthermore, the pair-wise antigenicity score between 21 HA proteins for both intra-groups including crystal-crystal (CC) and model-model (MM), and inter- group of crystal-model (CM) are calculated via CE-BLAST^3^. For two compared matrices, the Pearson correlation coefficients (PCCs) between corresponding rows are calculated and the averaged PCC score was used to measure the similarity between two matrices. Results showed that (**Supplementary** **Figure 1**), the PCC scores for compared matrices are 0.94 (CC-MM), 0.98 (MM-CM), and 0.95 (CM-CC), respectively. Above high similarity between both inter-groups and intra-groups indicated that structures constructed by homology modelling didn’t introduce extra variations and could be used for antigenicity measurement. Therefore, the antigen sequences in our benchmark dataset could be pre-constructed by homology modelling to obtain the conformational structures, and these structures could further be used to generate structure-based computational models for antigenicity measurement^3,4^.


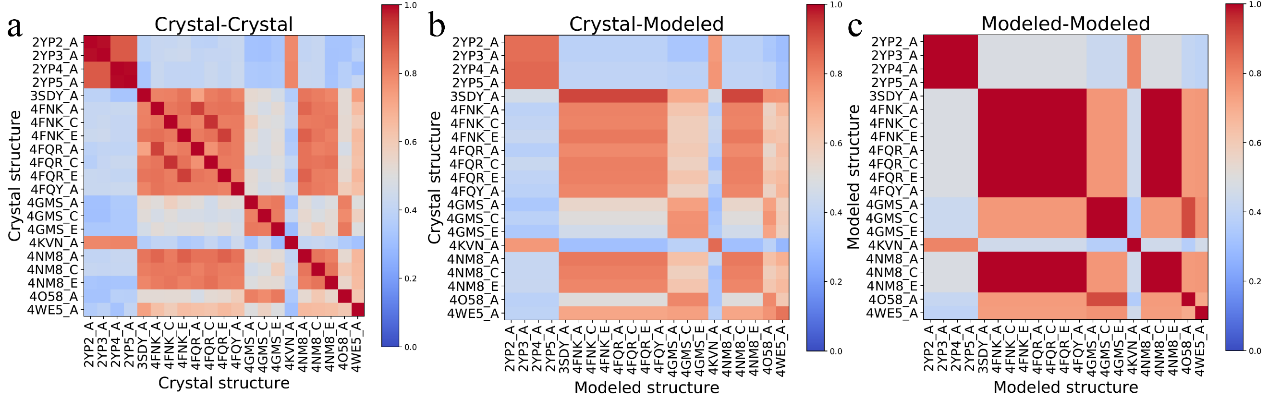


**Supplementary Figure 1.** Comparison of the antigenic distance for inter-groups and intra-groups of crystal structures and corresponding homology modelled structures. The color on each grid refers to the predicted CE-BLAST score of the corresponding pairs and marked with the gradient from blue (non-similar) to red (similar). (a) Each row and column represent a crystal structure. (b) Each row represents a crystal structure and each column represents a modelled HA1 structure. (c) Each row and column represent a modelled HA1 structure.

Reference:

1 Berman, H. M. et al. The Protein Data Bank. Nucleic Acids Res 28, 235-242, doi:10.1093/nar/28.1.235 (2000).

2 Eswar, N. et al. Comparative protein structure modeling using Modeller. Current protocols in bioinformatics / editoral board, Andreas D. Baxevanis ... [et al.] Chapter 5, Unit 5 6, doi:10.1002/0471250953.bi0506s15 (2006).

3 Qiu, T. et al. CE-BLAST makes it possible to compute antigenic similarity for newly emerging pathogens. Nature communications 9, 1772, doi:10.1038/s41467-018-04171-2 (2018).

4 Qiu, J. X., Qiu, T. Y., Yang, Y. Y., Wu, D. F. & Cao, Z. W. Incorporating structure context of HA protein to improve antigenicity calculation for influenza virus A/H3N2. Sci Rep-Uk 6 (2016).
